# The Effectiveness of Polypill for the Prevention of Cardiovascular Disease: A Meta-Analysis of Randomized Controlled Trials

**DOI:** 10.7759/cureus.47032

**Published:** 2023-10-14

**Authors:** Ghazala S Virk, Ashutosh Sharma, Momin R Khan, Krushi Shah, Jaina Mengar, Sandipkumar S Chaudhari, Saima Batool, Faraz Saleem

**Affiliations:** 1 Internal Medicine, Avalon University School of Medicine, Ohio, USA; 2 Internal Medicine, Kathmandu Medical College and Teaching Hospital, Kathmandu, NPL; 3 Internal Medicine, Khyber Medical College, Peshawar, PAK; 4 Internal Medicine, Gujarat Medical & Education Research Society (GMERS) Medical College, Gandhinagar, IND; 5 Medicine and Surgery, Government Medical College and New Civil Hospital, Surat, IND; 6 General Medicine, Lions General Hospital, Mehsana, IND; 7 Internal Medicine, Hameed Latif Hospital, Lahore, PAK; 8 Internal Medicine, California Institute of Behavioral Neurosciences & Psychology, Fairfield, USA; 9 Internal Medicine, Akhtar Saeed Medical and Dental College, Lahore, PAK

**Keywords:** randomized controlled trials, meta-analysis, cardiovascular disease, prevention, polypill, effectiveness

## Abstract

A significant global health concern, cardiovascular disease (CVD) is characterized by a rising prevalence and accompanying mortality rates. It is crucial to implement primary and secondary prevention strategies, particularly in resource-scarce settings. Polypills, which combine blood pressure, cholesterol, and homocysteine drugs, hold significant potential for lowering the risk of CVD. This study follows PRISMA meta-analysis guidelines. Two researchers conducted an extensive literature search. Inclusion criteria encompassed RCT design, polypill use, a four-week duration, and one meta-analysis outcome. Primary outcomes included MACE and CV mortality, while secondary outcomes encompassed SBP and LDL-C changes. Data extraction was performed independently, and conflicts were resolved. Review Manager 5.4 with random effects was employed for statistical analysis, and ROB 2.0 bias evaluation was conducted. The study reported CVD mortality and MACE risk ratios (RRs) with 95% CIs, as well as SBP and LDL-C weighted mean differences (MD). A total of 24 trials were included in this meta-analysis. The results revealed that the polypill was associated with a decreased risk of CVD mortality and major adverse cardiovascular events (MACE). Additionally, a significant reduction in systolic blood pressure (SBP) and low-density lipoprotein cholesterol (LDL-C) was observed. This meta-analysis showed that polypill is a viable medication for reducing the risk of CVD mortality and MACE. It is also a beneficial medication for lowering LDL-C levels and SBP.

## Introduction and background

Cardiovascular disease (CVD), which includes coronary artery disease, stroke, and peripheral arterial disease, continues to be the leading cause of substantial morbidity and mortality worldwide [1,2]. The prevalence of CVD among adults aged 20 years and older was documented at 127.9 million cases in 2020. This occurrence escalates with advancing age in both genders [3]. Cardiovascular-related deaths in the United States (U.S.) rose consistently from the 2010s to 2020 [4]. Projections suggest that this upward trend will persist through 2024 [5]. A survey demonstrated that over 50% of cardiovascular deaths might have been prevented by eradicating raised cholesterol levels, diabetes, hypertension, obesity, and smoking [6]. Hence, it is imperative to implement both primary and secondary preventive strategies to mitigate the widespread occurrence of CVD. While medication interventions for cardiovascular risk factors are recommended, their use for CVD prevention, particularly in resource-limited nations, remains suboptimal due to clinical inertia, patient adherence, and limited medication access, contributing to health disparities [7].

Fixed-dose combination (FDC) therapies, also known as polypills, represent a medication that combines multiple active pharmaceutical ingredients into complex formulations [8,9]. Proposed by Wald and Law, this method delivers active ingredients to reduce CVD risk in a single-dose form, including a blood pressure-lowering medication, a lipid-regulating medication, and a serum homocysteine-lowering medication, with or without the addition of an antiplatelet medication. It is recommended for all individuals aged 55 and above who do not have CVD (primary prevention) as well as those who have already been diagnosed with it (secondary prevention), irrespective of associated risk factors' assessment or severity [10]. Existing research on polypills is primarily composed of small-scale studies, often limited to specific nations. They typically concentrate on high-risk individuals for primary or secondary prevention, assessing outcomes over relatively short periods. Collectively, these studies demonstrate the benefits of the polypill approach [11]. Our analysis thus contributes by synthesizing and providing a broader perspective on this critical area of study.

Due to the scarcity of comprehensive long-term data regarding their efficacy in lowering CVD occurrences, FDCs are not currently accessible in the U.S. market [12]. The aim of our meta-analysis is to evaluate the impact of a polypill-based approach on blood pressure, cholesterol levels, and cardiovascular outcomes. Considering the widespread prevalence of CVD, our research provides essential strategies for combating this global health crisis.

## Review

Methods

The completion of this meta-analysis adhered to the requirements outlined by the Preferred Reporting Items for Systematic Reviews and Meta-Analyses (PRISMA) [13].

Search Strategy

Two writers independently conducted the literature search, data extraction, and quality assessments. We comprehensively searched Google Scholar, Cochrane Library, PubMed, Web of Science, Embase, and Scopus from inception until (September 15, 2023). The identified keywords for this study are "Polypill," "Polypills," and "Fixed-Dose Combination Pills," along with "cardiovascular disease prevention," "heart disease prevention," "hypertension," "hyperlipidemia," and "dyslipidemia." After screening titles and abstracts, potentially eligible studies were thoroughly reviewed in Table 1.

**Table 1:** Search stratergy

Database	Query	Search details	Results
PubMed	(Polypill) AND (cardiovascular disease prevention) AND (randomised control trial)	("polypill"[All Fields] OR "polypills"[All Fields]) AND (("cardiovascular diseases"[MeSH Terms] OR ("cardiovascular"[All Fields] AND "diseases"[All Fields]) OR "cardiovascular diseases"[All Fields] OR ("cardiovascular"[All Fields] AND "disease"[All Fields]) OR "cardiovascular disease"[All Fields]) AND ("prevent"[All Fields] OR "preventability"[All Fields] OR "preventable"[All Fields] OR "preventative"[All Fields] OR "preventatively"[All Fields] OR "preventatives"[All Fields] OR "prevented"[All Fields] OR "preventing"[All Fields] OR "prevention and control"[MeSH Subheading] OR ("prevention"[All Fields] AND "control"[All Fields]) OR "prevention and control"[All Fields] OR "prevention"[All Fields] OR "prevention s"[All Fields] OR "preventions"[All Fields] OR "preventive"[All Fields] OR "preventively"[All Fields] OR "preventives"[All Fields] OR "prevents"[All Fields])) AND (("random allocation"[MeSH Terms] OR ("random"[All Fields] AND "allocation"[All Fields]) OR "random allocation"[All Fields] OR "randomization"[All Fields] OR "randomized"[All Fields] OR "random"[All Fields] OR "randomisation"[All Fields] OR "randomisations"[All Fields] OR "randomise"[All Fields] OR "randomised"[All Fields] OR "randomising"[All Fields] OR "randomizations"[All Fields] OR "randomize"[All Fields] OR "randomizes"[All Fields] OR "randomizing"[All Fields] OR "randomness"[All Fields] OR "randoms"[All Fields]) AND ("controling"[All Fields] OR "controllability"[All Fields] OR "controllable"[All Fields] OR "controllably"[All Fields] OR "controller"[All Fields] OR "controller s"[All Fields] OR "controllers"[All Fields] OR "controlling"[All Fields] OR "controls"[All Fields] OR "prevention and control"[MeSH Subheading] OR ("prevention"[All Fields] AND "control"[All Fields]) OR "prevention and control"[All Fields] OR "control"[All Fields] OR "control groups"[MeSH Terms] OR ("control"[All Fields] AND "groups"[All Fields]) OR "control groups"[All Fields]) AND ("clinical trials as topic"[MeSH Terms] OR ("clinical"[All Fields] AND "trials"[All Fields] AND "topic"[All Fields]) OR "clinical trials as topic"[All Fields] OR "trial"[All Fields] OR "trial s"[All Fields] OR "trialed"[All Fields] OR "trialing"[All Fields] OR "trials"[All Fields]))	106
Embase			321
Cochrane Library			53
SCOPUS			257
Google Scholar			1134

Inclusion and Exclusion Criteria

Inclusion criteria encompassed a randomized controlled trial (RCT) design, the use of polypills in one comparison arm, a minimum study duration of four weeks, and at least one meta-analysis outcome. RCTs were included regardless of participants' cardiovascular disease state, whether they were undergoing primary or secondary prevention. The search was confined to English-language studies. Studies with multiple arms compared polypills to standard medication. If the standard therapy was unspecified, a placebo arm was included in the study design. Observational studies were excluded.

Measures of Outcomes and Data Extraction

Major adverse cardiovascular events (MACE) and cardiovascular death were the study's primary outcomes. The secondary outcomes of interest were changes in systolic blood pressure (SBP) measured in mmHg and low-density lipoprotein-cholesterol (LDL-C) assessed in mg/dl. Two authors independently retrieved published data and systematically arranged it in a table. The data extracted from research that met the predetermined criteria for inclusion was gathered using a standard method. The extracted data included the authors' names, publication year, sample size, details of the intervention, number of current smokers, baseline demographics (age, BMI, SBP, and LDL-C values), and clinical outcomes. Disagreements were resolved through consensus.

Statistical Analysis and Assessment of Bias

**Figure 1:**
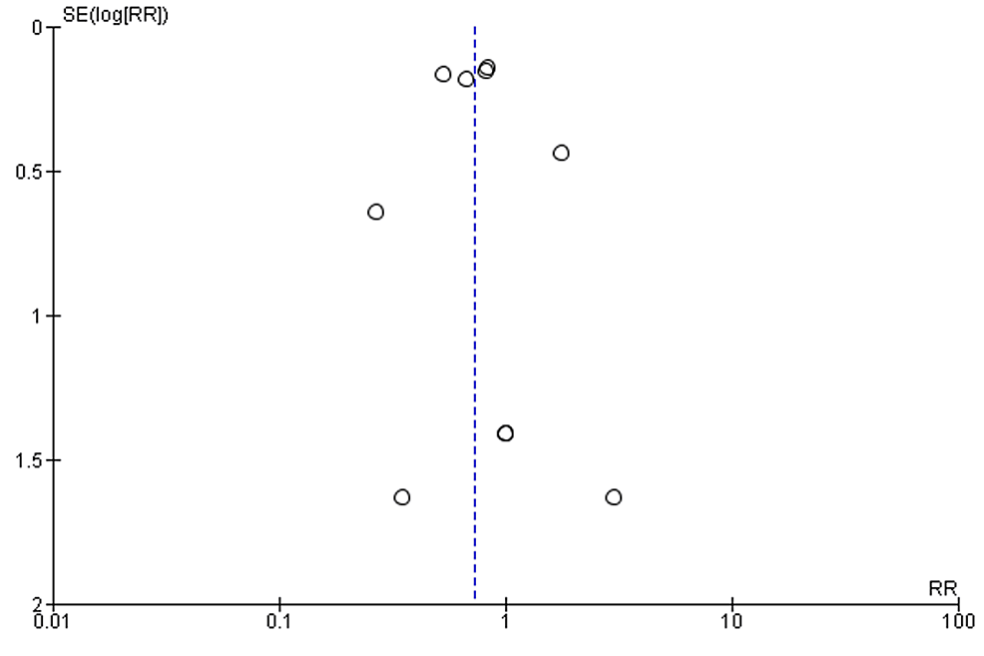
Funnel plot of comparison: 1 CVD; outcome: 1.1 CVD mortality.

**Figure 2:**
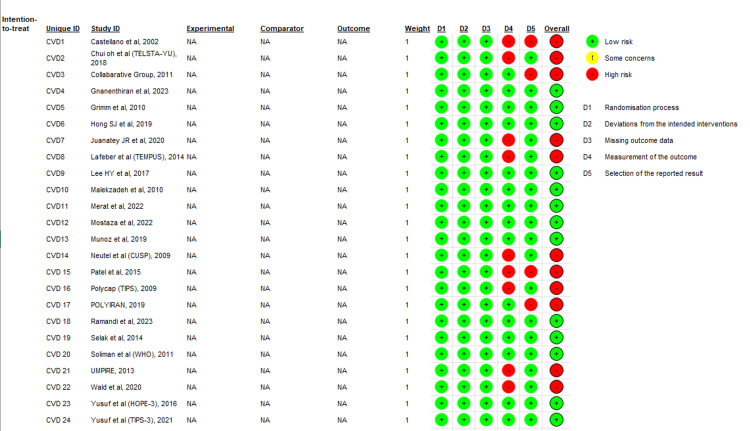
Quality assessment of the included studies. [15-38]

The statistical analysis and forest plots were prepared using Review Manager 5.4. Random effects were employed to aggregate effect values and account for study heterogeneity. The study provides an analysis of combined risk ratios (RRs) with their respective 95% confidence intervals (CIs) for the raw data, encompassing CVD mortality and MACE. Furthermore, the study presents weighted mean differences (MD) with their corresponding 95% confidence intervals (CIs) for continuous variables, such as changes in SBP and LDL-C. Publication bias was evaluated using a funnel plot, as illustrated in Figure 1, with a p-value below 0.05, indicating statistical significance. The quality assessment of clinical trials was conducted by two independent researchers. The Risk of Bias Tool 2 (ROB 2.0) [14] was utilized for this evaluation, as depicted in Figure 2. This approach is extensively employed in evaluating the quality of clinical trials.

Results

Study Selection

**Figure 3:**
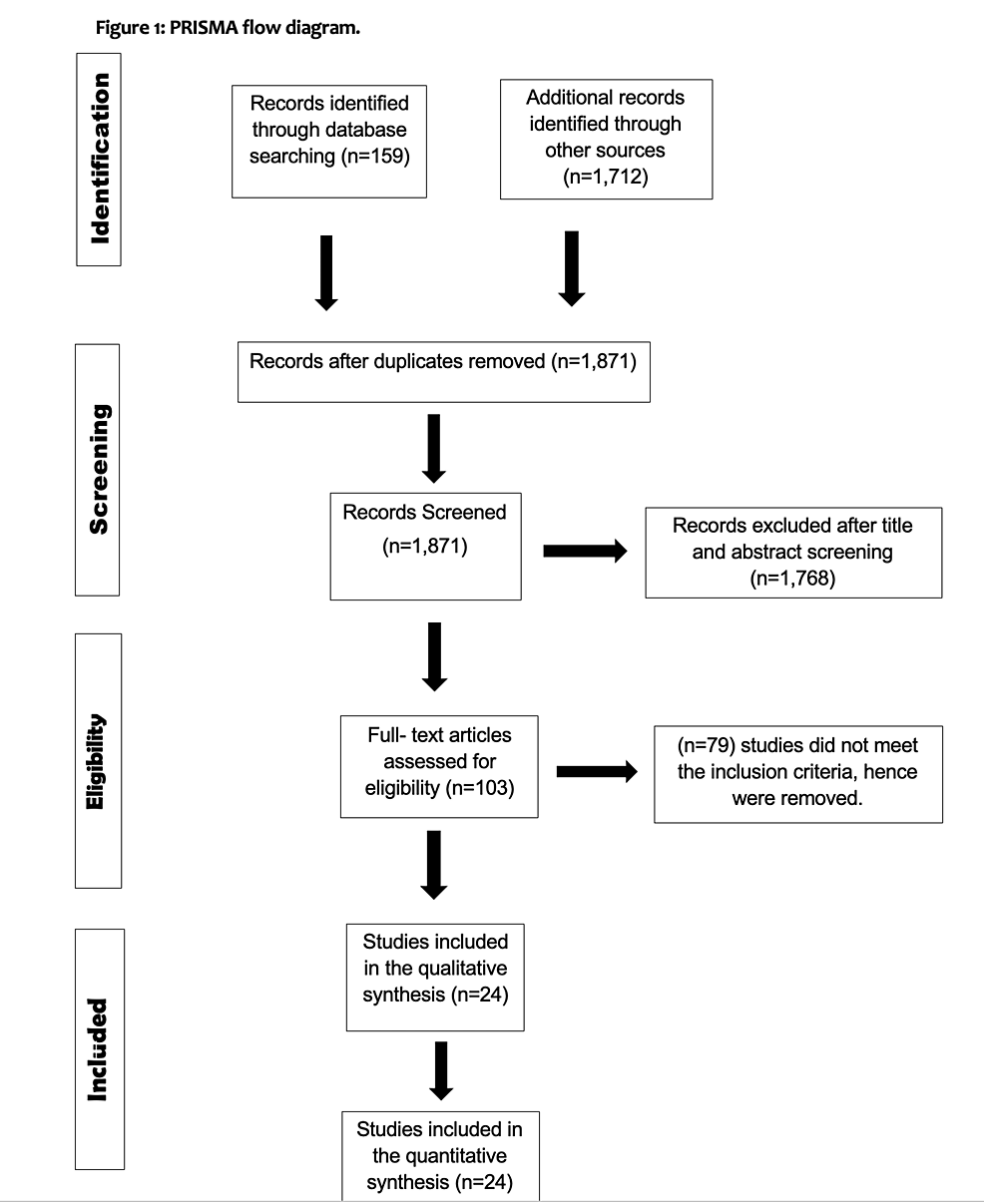
PRISMA flow diagram.

The procedure for selecting the studies is illustrated in Figure 3. Our preliminary search yielded 1871 studies. Records from 582 that were duplicated were discovered and deleted. One hundred and eight records were excluded due to their lack of relevance. The remaining 475 studies were chosen for further eligibility because of their relevance to the topic. Ultimately, 24 RCTs were chosen for the meta-analysis.

Baseline Characteristics of Included Studies

Table 2 shows the baseline characteristics of the included studies. The total number of patients in this study was 33,513, with a mean age of 60.9 years. About 55% of the patient population were males. The mean BMI of the patients was 27.8 kg/m2.

**Table 2:** Baseline Characteristics of the included studies. [15-38]

Author-Year	Sample size	Polypill ingredients, mg	Sex M/F	Smokers, n	Mean (SD)			
					Age, years	BMI, kg/m2	SBP, mmHga	LDL-C, mg/dL
Juanatey et al. 2020 [15]	321	Aspirin 100 atorvastatin 40 ramipril 10	176/145	N/A	58±10.2	29.7	138.7±12.7	137±33
Mostaza et al. 2022 [16]	439	Aspirin 100 Atorvastatin 20 or 40 Ramipril 2.5, 5.0 or 10	262/177	59	64.8	30.7	133.8	95.7
Castellano et al. 2022 [17]	2466	Aspirin 100 Ramipril 2.5, 5, or 10 Atorvastatin 40	1701/765	1265	76.0±6.6	N/A	129.1±17.7	89.2±37.2
Ramandi et al. 2023 [18]	1596	Aspirin 81 Hydrochlorothiazide 12.5 Atorvastatin 20 Valsartan 40	819/777	182	59 ± 6.7	28.9±4.5	133.8±21.7	125.4±33.9
Gnantherian et al. 2023 [19]	700	Telmisartan 20 Amlodipine 2.5 Chlorthalidone 12.5	297/403	34	56.2 ± 11.0	26±4.3	153.3±11.6	N/A
Merat et al. 2022 [20]	1508	Aspirin 81 Hydrochlorothiazide 12.5 Atorvastatin 20 Valsartan 40	772/736	317	60.8 ±6.6	28.1	133	N/A
Chul Oh et al. 2018 [21]	203	Telmisartan 80 Rosuvastatin 20	150/53	53	61.2	25.7	151	144
Hong S J et al. 2019 [22]	144	Telmisartan Amlodipine 80/10 + Rosuvastatin 20	33/111	N/A	66.8	26.9	147	154
Lafeber et al. 2014 [23]	78	Aspirin 75, Simvastatin 40, lisinopril 10, HCTZ 12.5	66/12	12	67	27.5	132	85
Lee H Y et al. 2017 [24]	143	Losartan 100 Amlodipine 5, Rosuvastatin 20	107/36	N/A	59.9	26.8	143	153.5
Munoz et al. 2019 [25]	303	Amlodipine 2.5, Atorvastatin 10, losartan 25, HCTZ 12.5	121/182	145	56	30.8	140	113
Patel et al. 2015 [26]	623	Aspirin 75 Simvastatin 40 Lisinopril 10 Atenolol 50 OR HCTZ 12.5	392/231	205	63.5	N/A	143	153.5
Roshandel et al. 2019 [27]	6838	Aspirin 81 Atorvastatin 20 HCTZ 12.5 Enalapril 5/valsartan 40	3398/3340	321	59.5	26.5	131	117.1
Thom et al. 2013 [28]	2004	Aspirin 75 Simvastatin 40 Lisinopril 10 Atenolol50 OR HCTZ 12.5	1642/362	275	61.8	27	137.4	91.5
Yusuf et al. 2021 [29]	5713	Aspirin 75 Simvastatin 40 Atenolol 100 HCTZ 25 Ramipril 10	2688/3025	512	63.9	25.8	144.5	120.7
Wald et al. 2012 [30]	84	Amlodipine 2.5 Losartan 25 HCTZ 12.5 Simvastatin 40	64/20	8	59	28	143	143
Selak et al. 2014 [31]	513	Aspirin 75 Simvastatin 40 Lisinopril 10 Atenolol 50 OR HCTZ 12.5	326/187	77	62	33	144	98.5
Malekzadeh et al. 2010 [32]	475	Aspirin 81 Atorvastatin 20 Enalapril 2.5 HCTZ 12.5	317/158	101	59	26.2	127.5	116
Neutel et al. 2009 [33]	123	Amlodipine 5 Atorvastatin 20	66/57	N/A	53	30.7	146.5	134
Rodgers et al. 2011 [34]	378	Aspirin 75 Simvastatin 20 Lisinopril 10 HCTZ 12.5	305/73	153	61.4	N/A	134	141
Yusuf et al. 2009 [35]	2053	Aspirin 100 Simvastatin 20 Ramipril 5 Atenolol 50 HCTZ 12.5	1152/901	276	54	26.3	134.4	16
Grimm et al. 2010 [36]	244	Amlodipine 5 Atorvastatin 10–20	123/121	74	56	N/A	132.6	129.5
Soliman et al. 2011 [37]	216	Aspirin 75 Simvastatin 20 Lisinopril 10 HCTZ 12.5	59/157	N/A	59.1	24.3	165.2	N/A
Yusuf et al. 2016 [38]	6348	Rosuvastatin 10 Candesartan 16, HCTZ 12.5	3405/2943	1780	65.7	27.1	138	127.5

Effect of intervention

CVD Mortality

**Figure 4:**
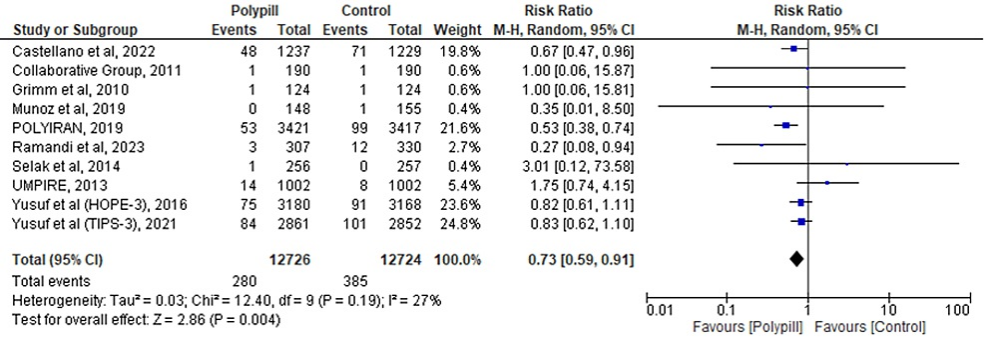
Forest plot comparing polypill versus control for the outcome of CVD mortality. [17-18,24-25,27,29,31,34,36-37]

Ten RCTs analyzed the effectiveness of the polypill versus control for the prevention of CVD. The results revealed that the polypill was significantly associated with a decreased risk of CVD mortality when compared to standard care (RR = 0.73; 95% CI: 0.59-0.91, p < 0.00001; I2 = 27%) (Figure 4).

MACE

**Figure 5:**
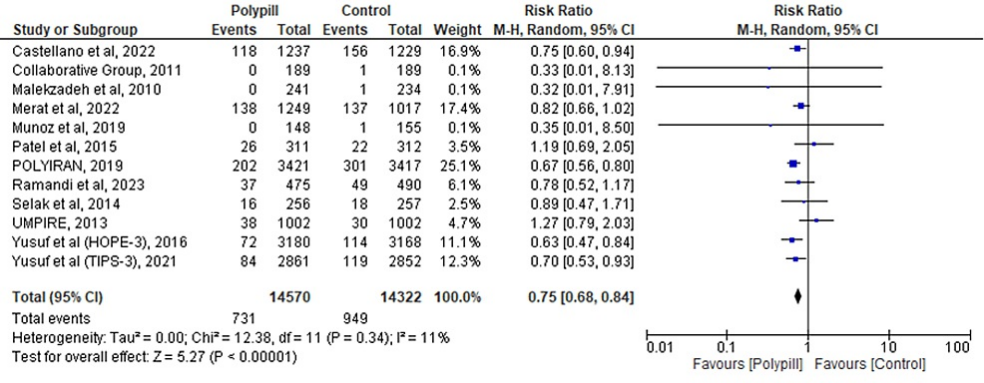
Forest plot comparing polypill versus control for the outcome of MACE. [17-18,20,25-29,31-32,34,38]

Twelve studies evaluated the efficacy of the polypill versus the control on MACE. The findings demonstrated a significant reduction in the risk of MACE in the group of patients taking the polypill when compared to standard care (RR = 0.75; 95% CI: 0.68-0.84; p = 0.004; I2 = 11%) (Figure 5).

Change in SBP

**Figure 6:**
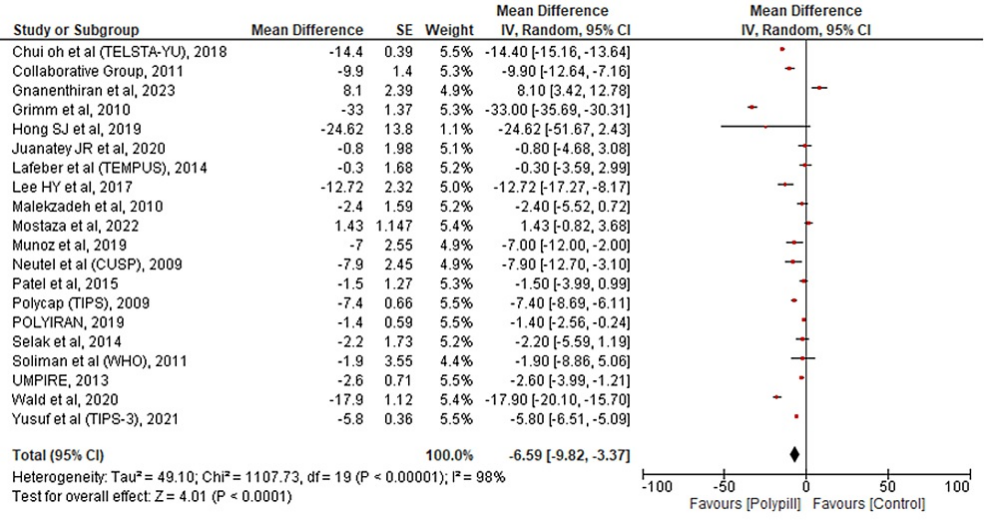
Forest plot comparing polypill versus control for the outcome of the change in SBP. [15-16,19,21-37]

The findings of 20 studies were pooled to assess the change in SBP in the two groups, namely polypill and control. It was revealed that there was a significant reduction in SBP, favoring the polypill group over standard care (MD = -6.59; 95% CI: -9.82--3.37; p < 0.0001; I2 = 98%) (Figure 6).

Change in LDL-C

**Figure 7:**
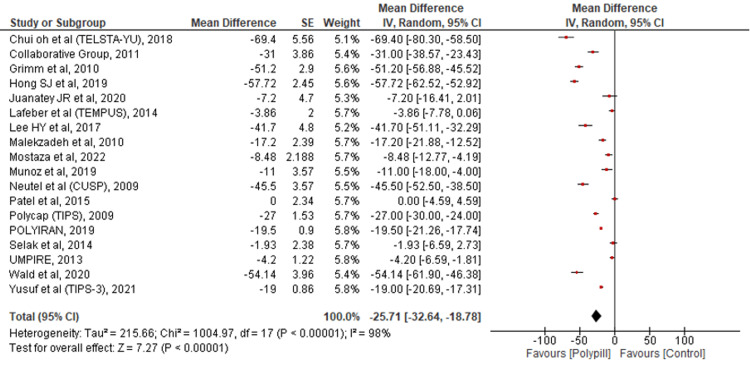
Forest plot comparing polypill versus control for the outcome of change in LDL-C. [15-16,21-36]

To evaluate the change in LDL-C, findings from 18 studies were pooled together. The results showed that there was a significant reduction in LDL-C, favoring the polypill group over standard care (MD = -25.71; 95% CI: -32.64--18.78; p < 0.00001; I2 = 98%) (Figure 7).

Discussion

In this meta-analysis aimed at determining the impact of the polypill on cardiovascular disease (CVD) outcomes compared to standard care in patients with or without a history of CVD, we observed that the polypill improved the outcomes of CVD mortality and major adverse cardiovascular events (MACE). Additionally, the polypill was associated with a significant reduction in systolic blood pressure (SBP) (mmHg) and low-density lipoprotein cholesterol (LDL-C) (mg/dl), making it an effective medication for lowering blood pressure and controlling lipid levels in the body.

Several previous meta-analyses have explored the effect of the polypill on CVD outcomes. For example, Abushouk et al. [39] demonstrated a significant decrease in MACE risk in studies targeting primary prevention exclusively. However, this study did not reveal an overall effect of the polypill on the reduction of MACE risk (RR=0.84; 95% CI=0.68-1.04) and CVD mortality (RR=0.90; 95% CI=0.79-1.01). Similarly, a study by Hennawi et al. [40] showed that polypill therapy was associated with a statistically significant reduction in SBP (OR: -0.33, 95% CI [-0.64, -0.03]; P-value= 0.03) and total cholesterol level (OR: -1.25, 95% CI [-1.82, -0.68]; P= 0.0001). However, it did not find statistically significant benefits in terms of all-cause mortality, MACE, and LDL-C levels. In contrast, our meta-analysis demonstrated improvements in all these outcomes.

Another meta-analysis by Mohamed et al. [12] reported results consistent with the findings of our meta-analysis. This study showed that the polypill was associated with decreased SBP (Mean Difference [MD] -6.39; [95% CI -9.21,-3.56]; p<0.001), LDL-C (MD -27.92, [95% CI -35.39, -20.44]; p<0.001), CVD mortality (RR= 0.78; 95% CI= 0.61-0.99); P= 0.04), and MACE (RR= 0.76; 95% CI= 0.64-0.91); P = 0.002]. However, this meta-analysis included only 18 RCTs, whereas our study encompassed 24 articles, thus enhancing the robustness of these findings.

Metabolic factors, such as hypertension and hyperlipidemia, have been found to have a substantial impact on the risk of cardiovascular disease (CVD) [41,42]. Blood pressure and cholesterol levels, with blood pressure playing a prominent role as the principal mediator, influence the risk of CVD [43]. Additionally, a strong and persistent association exists between cardiovascular risk factors (hypertension and dyslipidemia) and CVD mortality [44]. The need for combination therapy with multiple medications arises from the clustering of various risk factors. However, implementing complex treatment plans may lead to suboptimal compliance and subsequently result in negative outcomes [45].

The use of the polypill in clinical practice and research for the prevention and treatment of cardiovascular outcomes has significant implications. From a clinical perspective, the polypill offers a streamlined method of controlling cardiovascular risk by simplifying prescription schedules for patients with multiple risk factors. This approach enhances medication compliance, which is crucial for achieving the most effective cardiovascular outcomes. Furthermore, it provides a compelling option for both primary and secondary prevention measures due to its potential to lower LDL-C, reduce blood pressure, decrease the risk of MACE and CVD mortality, and lower SBP.

The polypill offers researchers a viable means to examine the comprehensive management of cardiovascular health. Its success in improving various cardiovascular markers raises the possibility that it may serve as a valuable tool for planning clinical trials that investigate cutting-edge approaches to CVD prevention and therapy. Additionally, future studies on the long-term effectiveness and safety of polypill therapy will help us understand its role in cardiovascular care. Moreover, with the potential to enhance patient adherence and reduce the burden of cardiovascular disease, the use of the polypill represents a significant advancement in cardiovascular medicine. It offers clinicians and researchers a comprehensive and straightforward approach to managing and studying cardiovascular outcomes.

Limitations

Our research is subject to certain inherent limitations. Our secondary outcomes exhibited significant heterogeneity, as evidenced by the various methodologies employed and the range of reported outcomes. The length of the follow-up period varied significantly, ranging from 6 weeks to 65 months, and several studies had divergent standards of care and behaviors. Furthermore, our analysis relied on summary-level data instead of individual-level data, necessitating a cautious approach to interpreting the findings.

## Conclusions

This meta-analysis revealed that polypills were associated with significantly lower levels of SBP and LDL-C compared to standard treatment. Additionally, it showed a noticeable reduction in major adverse cardiovascular events (MACE) and cardiovascular disease (CVD) mortality. These effects were observed consistently in both patients with and without pre-existing CVD. In terms of clinical outcomes, polypills demonstrated comparability to the standard of care, with improved adherence and no significant safety concerns. Further research is necessary, especially focusing on populations with limited treatment adherence.
